# Supplementary figures and images for: Antiproliferative activity of Syzygium coriaceum, an endemic plant of Mauritius, with its UPLC-MS metabolite fingerprint: A mechanistic study
Source: PLoS One. 2021 Jun 1;16(6):e0252276. doi: 10.1371/journal.pone.0252276 (PMC8168845; doi:10.1371/journal.pone.0252276)

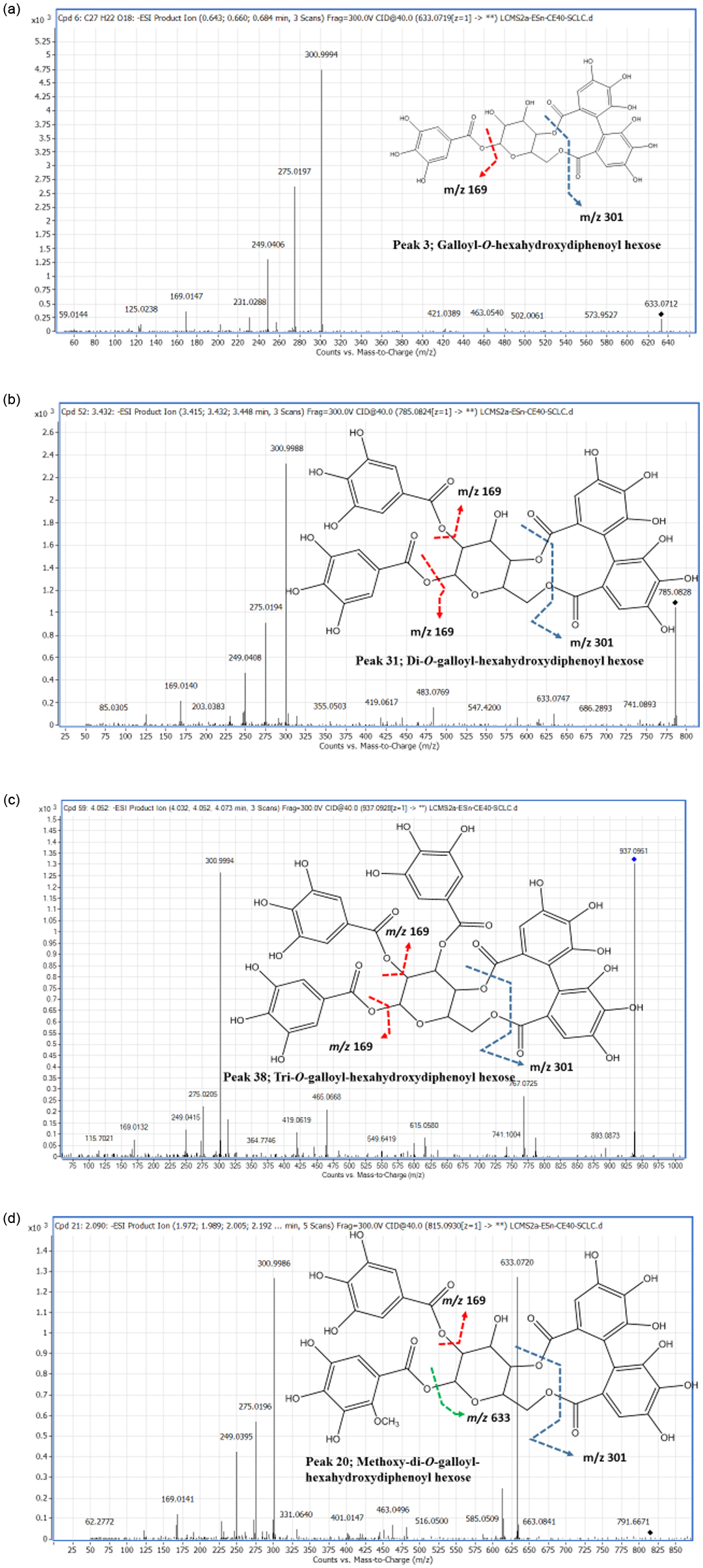

Supplement: S1 Fig — a. ESI-MS/MS spectrum of galloyl-O-hexahydroxydiphenoyl hexose (peak 3; C27H21O18−) in the negative ion mode. b. ESI-MS/MS spectrum of di-O-galloyl-hexahydroxydiphenoyl hexose (peak 31; C34H25O22−) in the negative ion mode. c. ESI-MS/MS spectrum of tri-O-galloyl-hexahydroxydiphenoyl hexose (peak 38; C41H29O26−) in the negative ion mode. d. ESI-MS/MS spectrum of methoxy-di-O-galloyl-hexahydroxydiphenoyl hexose (peak 20; C35H27O23−) in the negative ion mode. (TIF) [file pone.0252276.s001.tif]

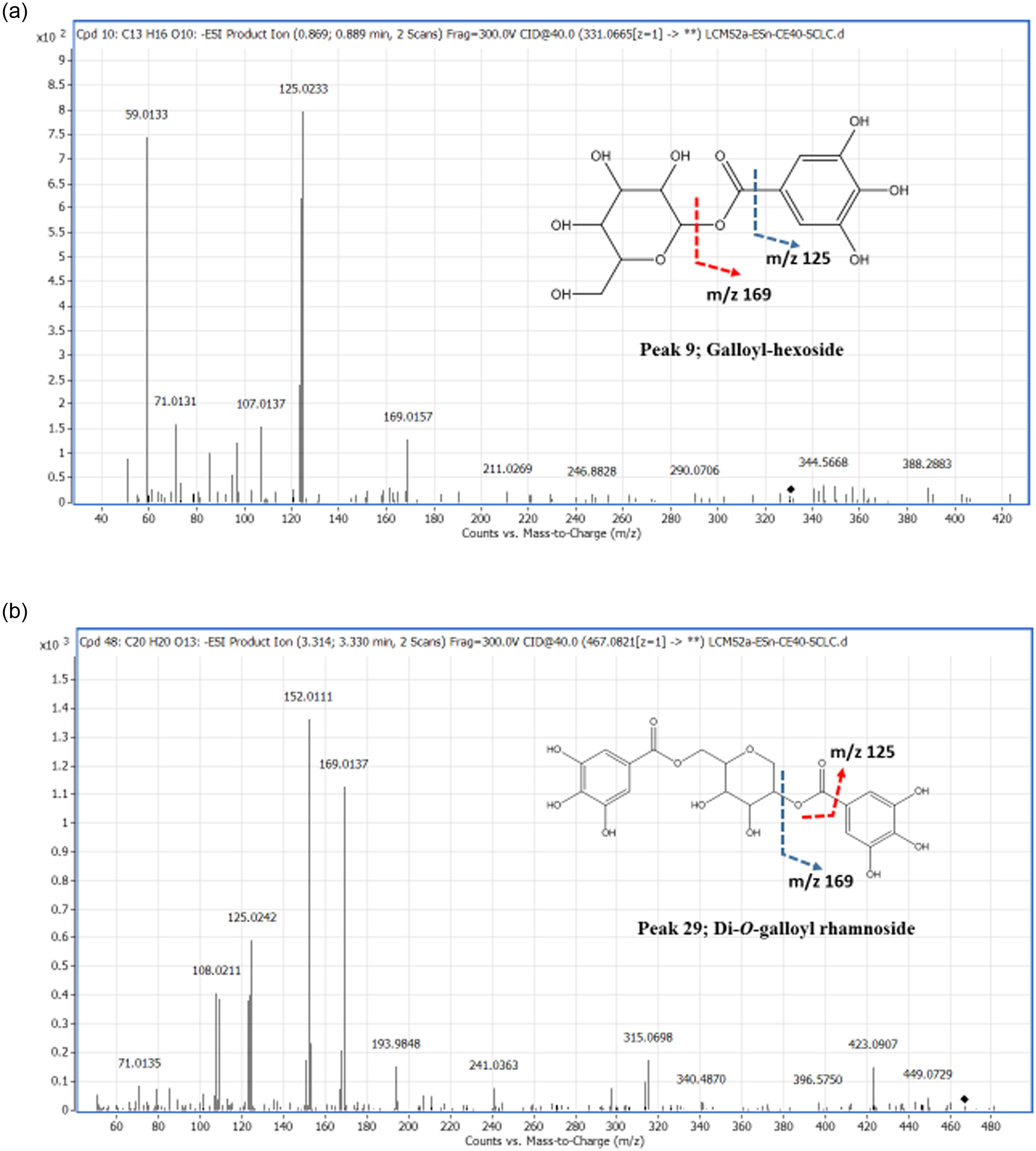

Supplement: S2 Fig — a. ESI-MS/MS spectrum of galloyl-O-hexoside (peak 9; C13H15O10−) in the negative ion mode. b. ESI-MS/MS spectrum of di-O-galloyl rhamnoside (peak 29; C20H19O13−) in the negative ion mode. (TIF) [file pone.0252276.s002.tif]

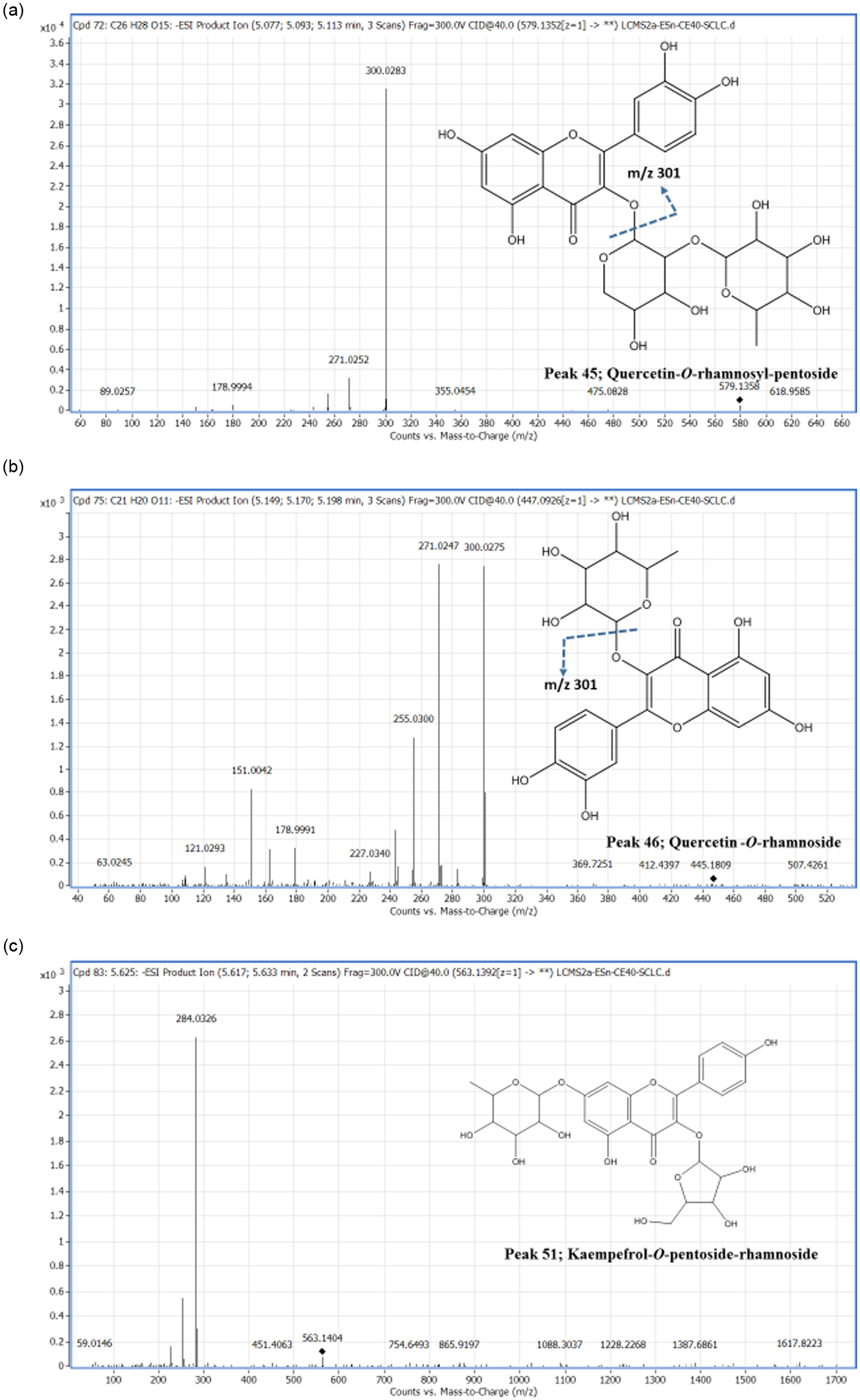

Supplement: S3 Fig — a. ESI-MS/MS spectrum of quercetin-O-pentosly-rhamnoside (peak 45; C26H27O15−) in the negative ion mode. b. ESI-MS/MS spectrum of quercetin-O-rhamnoside (peak 46; C21H19O11−) in the negative ion mode. c. ESI-MS/MS spectrum of kaempefrol-O-pentosyl-rhamnoside (peak 51; C26H27O14−) in the negative ion mode. (TIF) [file pone.0252276.s003.tif]

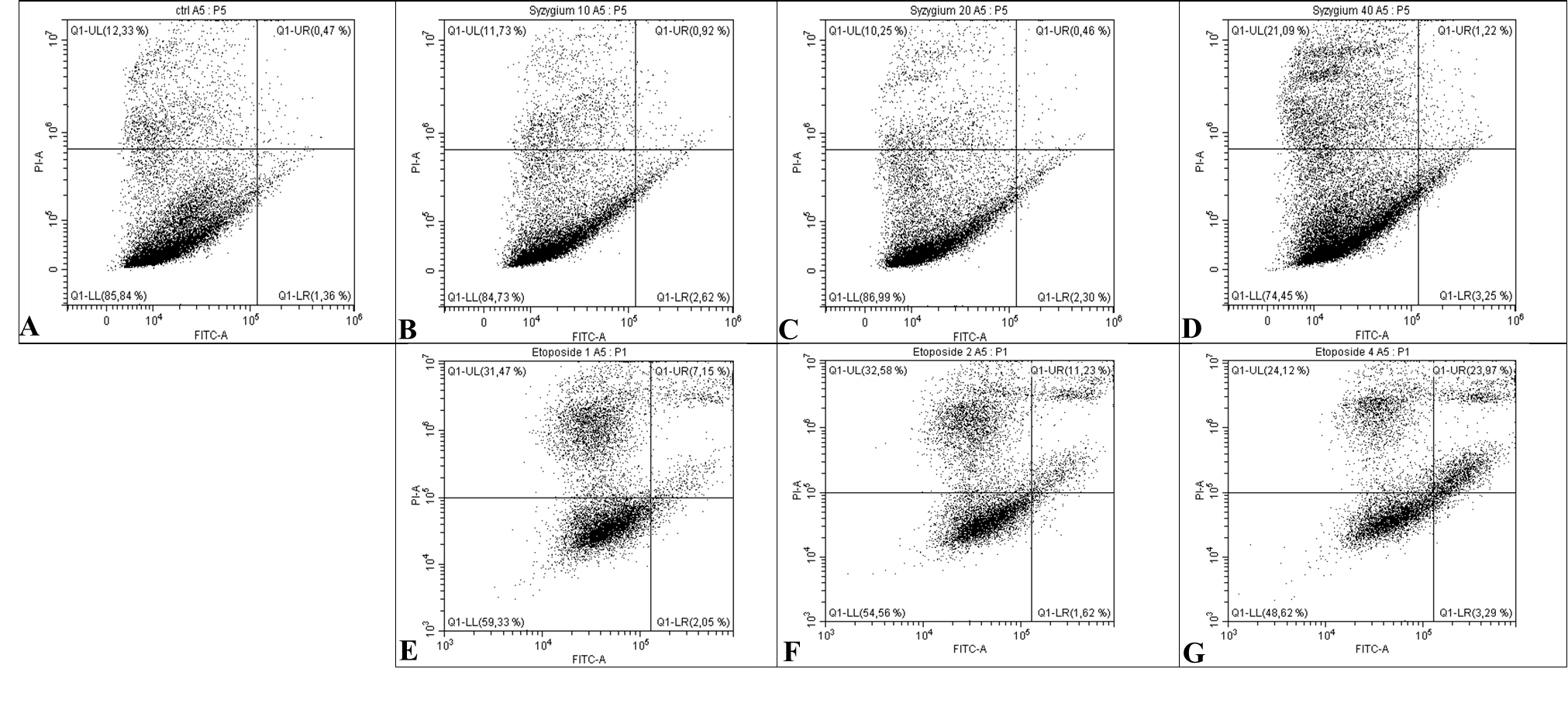

Supplement: S4 Fig — Cells were treated for 48 hours with (A) negative control (0.025% DMSO); (B, C, D) 10 μg/ml, 20 μg/ml and 40 μg/ml of Syzygium coriaceum, respectively; (E, F, G) and 1 μg/ml, 2 μg/ml and 4 μg/ml of etoposide, respectively. (TIF) [file pone.0252276.s004.tif]

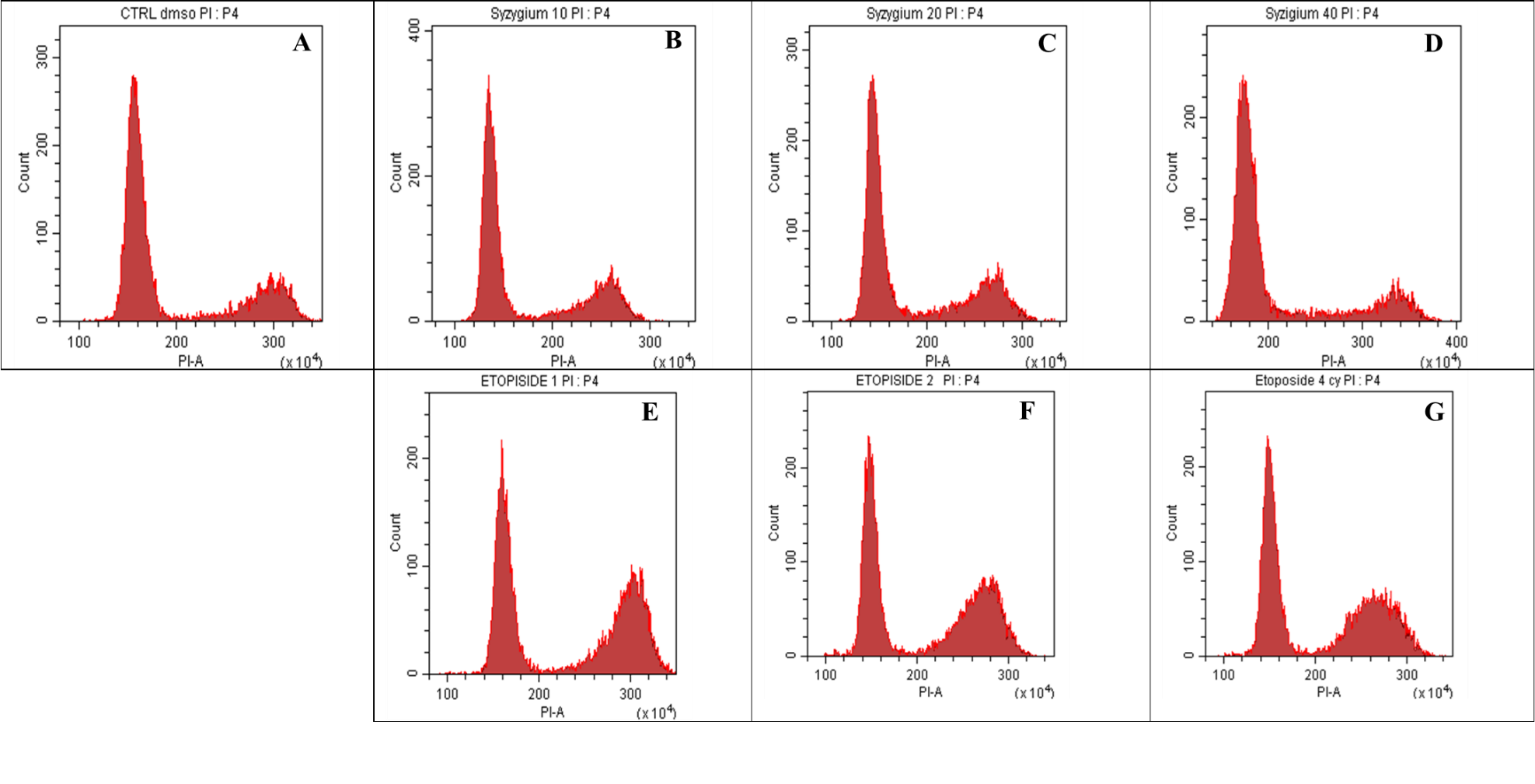

Supplement: S5 Fig — Cells were treated for 48 hours with (A) negative control (0.025% DMSO); (B, C, D) 10 μg/ml, 20 μg/ml and 40 μg/ml of S. coriaceum, respectively and (E, F, G) 1 μg/ml, 2 μg/ml and 4 μg/ml of etoposide, respectively. (TIF) [file pone.0252276.s005.tif]

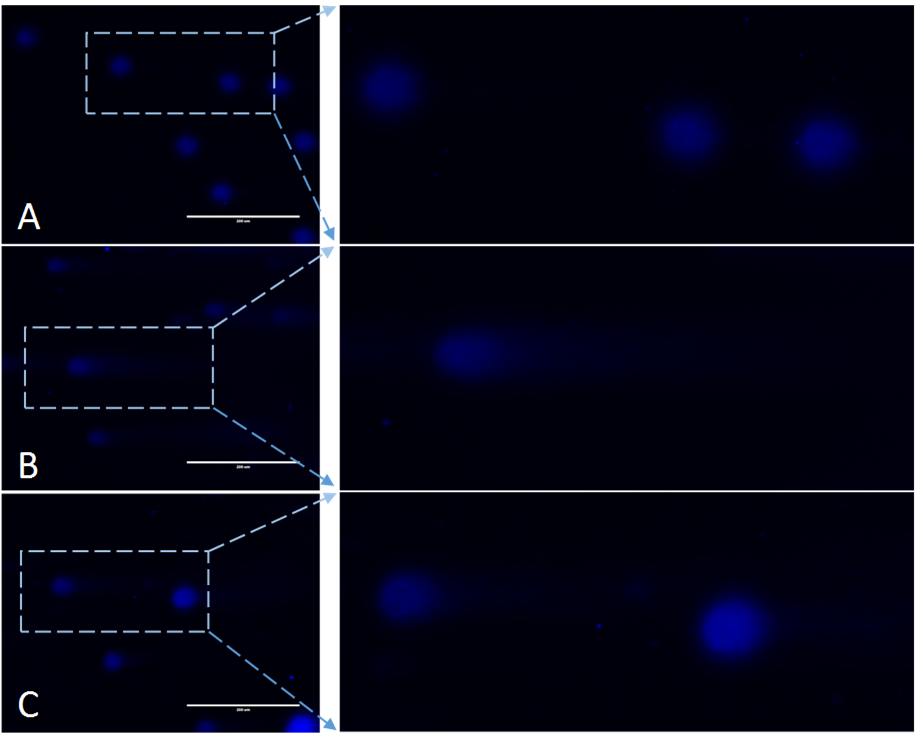

Supplement: S6 Fig — DNA fragments in HepG2 cells after 24 hours exposure to (A) cell culture medium (negative control); (B) 200 μM H2O2 (positive control; 30 minutes) and (C) 10 μg/ml Syzygium coriaceum extract. Each figure represents a typical comet tail of the 100 observed cells from each experiment; magnification 200X, (n = 3). (TIF) [file pone.0252276.s006.tif]
