# Supplementary material for: Antiproliferative activity of Syzygium coriaceum, an endemic plant of Mauritius, with its UPLC-MS metabolite fingerprint: A mechanistic study
Source: PLoS One. 2021 Jun 1;16(6):e0252276. doi: 10.1371/journal.pone.0252276 (PMC8168845; doi:10.1371/journal.pone.0252276)
Supplement: Attachment — Submitted filename: Response to Reviewers 2-PLOS.docx [file pone.0252276.s008.docx]

The point-to-point response to the reviewer’s comments is provided below.

The reviewer comments are given in italics font and the authors response are given in bold font.

*The text still contains typographic errors which have to be resolved before accepting for publication. Moreover, language usage should be further polished in orded to improve general readability of the manuscript. Please do not use random word capitalization in both the body text and subtitles.*

**The typographic errors have been resolved and the readability of the manuscript has been checked. Random word capitalization has been deleted both in text and in subtitles.**

*L172: Please define "100 U/L streptomycin-penicillin".*

**Line 176 now reads as “100 units /L penicillin and 100 units /L streptomycin”.**

*L225: "Flow cytometry analysis" should stand instead.*

**Line 231 now reads as “Flow cytometry analysis”**

*L250: Metabolite fingerprint*

**Line 231 now reads as “Metabolite fingerprint of *S. coriaceum* leaf extract”**

*Table 1: "Hexahydroxydiphenoylproto-quercitoL", "L" stands in upper case.*

"Hexahydroxydiphenoylproto-quercitoL" in table 1 now reads as “Hexahydroxydiphenoylproto-quercitol”.

*L319-335: Please do not capitalize random words or compound names unless they stand at the beginning of a sentence.*

Lines 324 to 347 are revised to now read as follows:

**“Fig 3: (A) Molecular network group of hexahydroxydiphenoyl hexose derived compounds. Peak numbers are according to that listed in (Table 1) as follows: peak 3; galloyl-*O*-hexahydroxydiphenoyl hexose, peak 20; methoxy-di-*O*-galloyl-hexahydroxydiphenoyl hexose, peak 22; ethoxygalloyl-*O*-hexahydroxydiphenoyl hexose, peak 31; di-*O*-galloyl-hexahydroxydiphenoyl hexose, peak 35; methoxy-tri-*O*-galloyl-hexahydroxydiphenoyl hexose, peak 38; tri-*O*-galloyl-hexahydroxydiphenoyl hexose, peak 42; methoxy-tetra-*O*-galloyl-hexahydroxydiphenoyl hexose. (B) Molecular network group of sugar polygalloyl esters. Peak numbers are according to that listed in (Table 1) as follows: peak 14; di-*O*-galloyl-hexoside, peak 24; tri-*O*-galloyl-hexoside, peak 32; tetra-*O*-galloyl-hexoside, peak 43; penta-*O*-galloyl-hexoside. (C) Molecular network group of amino alcohols measured under positive ionization mode. peak numbers are according to that listed in (Table 1) as follows: peak 55; amino-tetradecanediol, peak 56; amino-hexadecanediol, peak 58; amino-octadecanetriol, peak 60; amino-heptadecanediol, peak 61; amino-octadecanediol, peak 63; aminoeicosanediol, peak 64; aminodocosanediol. Nodes are color-coded in the three networks according to their *m/z* ranges and labelled by their parent ions. Edges are labelled by the mass differences between each node pair where their line widths are related to the cosine score (from thin, cosine score: 0.7, to thick, cosine score: 1).”**
